# Evolutionary Relationships Between Dysregulated Genes in Oral Squamous Cell Carcinoma and Oral Microbiota

**DOI:** 10.3389/fcimb.2022.931011

**Published:** 2022-07-13

**Authors:** Yang Fang, Yi Yang, Chengcheng Liu

**Affiliations:** ^1^ Department of Laboratory Medicine, Third Affiliated Hospital of Zhengzhou University, Zhengzhou, China; ^2^ State Key Laboratory of Oral Diseases, National Clinical Research Center for Oral Diseases, Department of Periodontics, West China School and Hospital of Stomatology, Sichuan University, Chengdu, China

**Keywords:** OSCC, oral microbiota, evolutionary relationships, matrix metalloproteinases, DEGs1

## Abstract

Oral squamous cell carcinoma (OSCC) is one of the most prevalent cancers in the world. Changes in the composition and abundance of oral microbiota are associated with the development and metastasis of OSCC. To elucidate the exact roles of the oral microbiota in OSCC, it is essential to reveal the evolutionary relationships between the dysregulated genes in OSCC progression and the oral microbiota. Thus, we interrogated the microarray and high-throughput sequencing datasets to obtain the transcriptional landscape of OSCC. After identifying differentially expressed genes (DEGs) with three different methods, pathway and functional analyses were also performed. A total of 127 genes were identified as common DEGs, which were enriched in extracellular matrix organization and cytokine related pathways. Furthermore, we established a predictive pipeline for detecting the coevolutionary of dysregulated host genes and microbial proteomes based on the homology method, and this pipeline was employed to analyze the evolutionary relations between the seven most dysregulated genes (MMP13, MMP7, MMP1, CXCL13, CRISPO3, CYP3A4, and CRNN) and microbiota obtained from the eHOMD database. We found that cytochrome P450 3A4 (CYP3A4), a member of the cytochrome P450 family of oxidizing enzymes, was associated with 45 microbes from the eHOMD database and involved in the oral habitat of *Comamonas testosteroni* and *Arachnia rubra*. The peptidase M10 family of matrix metalloproteinases (MMP13, MMP7, and MMP1) was associated with *Lacticaseibacillus paracasei*, *Lacticaseibacillus rhamnosus*, *Streptococcus salivarius*, *Tannerella* sp.*_HMT_286*, and *Streptococcus infantis* in the oral cavity. Overall, this study revealed the dysregulated genes in OSCC and explored their evolutionary relationship with oral microbiota, which provides new insight for exploring the microbiota–host interactions in diseases.

## Introduction

Head and neck squamous cell carcinoma (HNSCC) is the seventh most common malignancy in the world, accounting for more than 90% of head and neck malignancies (Mody et al., 2021). HNSCC originates from the squamous epithelium of the upper respiratory tract and digestive tract of the oral cavity, pharynx, and larynx, among which pharyngeal squamous cell carcinoma, laryngeal squamous cell carcinoma, and OSCC are the most common. OSCC often has a great impact on patients’ chewing, swallowing, language, breathing, and other functions and even threatens their lives (Johnson et al., 2020). In recent years, the incidence of OSCC has been on the rise, becoming a world public health problem with high morbidity and mortality (Ferlay et al., 2019). Studies have shown that, in addition to major risk factors such as tobacco and alcohol abuse, exposure to environmental pollutants and viruses, specific oral bacteria, or oral microbial communities may play an important role in the occurrence and progression of OSCC (Fitzsimonds et al., 2020; Irfan et al., 2020). The human microbiome coevolved and coexisted, and OSCC that also grows in the oral cavity may themselves be the hosts of oral microbiota. The oral cavity harbors over 700 microbial species and both pathogenic and commensal strains are involved in the development of OSCC. Evidence has indicated a correlation of some specific species with OSCC, including *Porphyromonas gingivalis*, *Fusobacterium nucleatum*, *Treponema denticola*, *Streptococcus gordonii*, and human papilloma virus 16 (Fitzsimonds et al., 2020).

The current strategies to investigate the role of the oral microbiota in OSCC have predominantly focused on detecting oral microbial communities present or populational shifts in OSCC samples and studying the effect and mechanism of specific oral microbial challenges on biological processes (BPs) related to OSCC occurrences, such as cell proliferation, cell apoptosis, and the epithelial to mesenchymal transition. Perspective studies should focus on exploring the oral microbiota potentially related to OSCC. However, oral microbes are abundant and approximately 30% of them cannot be cultured. Thus, determining oral microbial–host interactions between species experimentally is a challenging task (Fritz et al., 2013). Computational approaches are an ideal approach to aid in screening for microbial–host interactions, with time-saving and economic advantages (Dix et al., 2016; Fitzsimonds et al., 2020). From an evolutionary perspective, if there is significant similarity between two protein sequences, they may originate from a common ancestor and have the same or similar functions (Pearson, 2013). Therefore, the most common method to explore protein function is pair-wise protein sequence comparison to “transfer” or prediction of function based on sequence similarity between proteins of known and unknown function (Rost, 1999; Bork, 2000; Devos and Valencia, 2000). And BLAST is a classic pairwise approach that can search protein sequence similarities all against all (Camacho et al., 2009). Based on this principle, several approaches have been established to determine pathogen–host protein–protein interactions (PHIs), including protein homology prediction, structural domain–based methods, and machine learning–based methods (English and Albersheim, 1969; Wojcik and Schächter, 2001; Pagel et al., 2004; Qi et al., 2010; Dyer et al., 2011).

Increasing evidence has shown that shared evolutionary history matters to both microbiota and hosts (Davenport et al., 2017). Microorganisms include bacteria, viruses, fungi, and some small protists. These organisms typically have smaller proteomes, based on which they can be analyzed for community heterogeneity, activity, and function. If the entire proteome of a microorganism is regarded as a protein that is evolutionarily conserved, the entire microbiome is a living organism composed of a large number of such proteins. Based on the principle of protein–protein interaction (PPI), the interaction relationship between the entire proteome of microorganisms and the host can be predicted. Oral microbiota colonize the oral mucosa, and they have a coevolutionary relationship; therefore, based on the principle of coevolutionary association inferring functional interactions, this study established a predictive pipeline for the evolutionarily interconnected evolution of dysregulated host genes and microbial proteomes (Devos and Valencia, 2000). We identified dysregulated genes in OSCC tissues by analyzing gene expression datasets from the GEO database and explored their evolutionary relations with oral microbiota with this pipeline. The results are expected to provide new insights into the interactions between oral microbiota and OSCC.

## Materials and methods

### Data Collection

The Gene Expression Omnibus (GEO) database contains a large number of gene expression profiling (high-throughput sequencing and microarray datasets) and RNA methylation profiles that are submitted by different research laboratories in the world (Edgar et al., 2002). We retrieved the related gene expression datasets by OSCC and oral keywords. The criteria for the retrieved datasets must contain different sequencing platforms. Finally, the microarray datasets (GSE138206) and high-throughput sequencing datasets (GSE140707) were downloaded from the GEO database. The GSE138206 dataset contains six OSCC tissues (Ca), tissues adjacent to cancer (P), and contralateral normal tissues (N), and the GSE140707 contains three tumorous and adjacent tissues from OSCC sufferers.

### DEGs Identification

All analyses of differentially expressed genes (DEGs) were performed by R language. The GEO query package (Sean and Meltzer, 2007) was used to obtain collected datasets from the GEO database. For microarray datasets, the statistically significant DEGs were acquired by utilizing the limma package (Ritchie et al., 2015) with adjusted P-value (FDR) < 0.05 and |log2 (fold change (FC))| > 1. The Deseq2 (Love et al., 2014) and EdgeR (Robinson et al., 2010) packages were utilized to analyze RNA sequencing data and filter significant DEGs between OSCC and adjacent normal tissues.

### Enrichment Analyses

The Gene Ontology (GO) describes our knowledge of the biological function in three aspects: molecular function (MF), cellular component (CC), and BP (The Gene Ontology, Consortium 2019). The Kyoto Encyclopedia of Genes and Genomes (KEGG) pathway is a database utilized for genomic and biological pathway and other omics studies (Kanehisa and Goto, 2000). We used the ClusterProfile package (Yu et al., 2012) to identify the potential functions of the significant DEGs with the GO and KEGG databases, and the results were displayed by the ggplot2 package (Wickham, 2011) with a cutoff of p < 0.05.

### Evolutionary Relation With the Oral Microbiome

The microbe data (1,903 microbiome genomes with 4,665,857 proteins) were downloaded from the expanded Human Oral Microbiome Database (eHOMD) (Escapa et al., 2018). The eHOMD provides comprehensive curated information on bacteria in the human mouth and aerodigestive tract, including the pharynx, nasal passages, sinuses, and esophagus. The DEG homologous proteins were searched by BLAST+ software (Camacho et al., 2009) with an e-value < 0.001. We used the MEGA11 software to identify the evolutionary relationship between DEGs and oral microbiomes by MEGA11 software (Tamura et al., 2021). Multiple sequence alignment was performed by MUSCLE (Edgar, 2004) (Gap Open:-2.9, Hydrophobicity Multiplier:1.2, Max Iterations:16, Min Diag Length:24), using the maximum likelihood method (JTT model and NNI ML heuristic method) to reconstruct the phylogenetic tree. The iTOL is used to illustrate the phylogenetic tree (Letunic and Bork, 2006).

## Results

### Transcriptional Landscapes of OSCC

**Figure 1:**
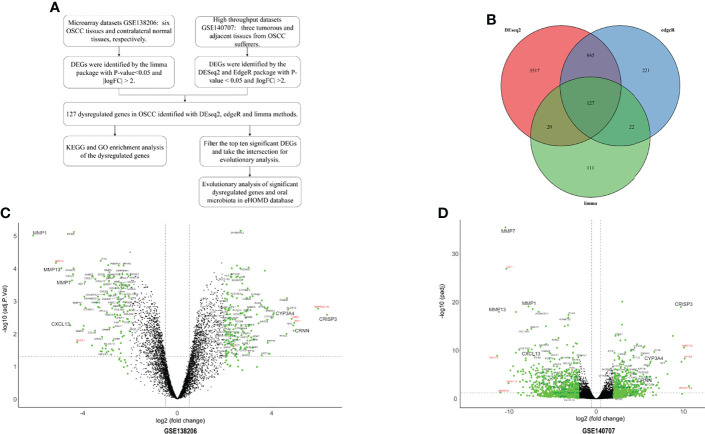
The identified of DEGs. **(A)** The flowchart of research design. **(B)** The two datasets showed an overlap of 127 differentially expressed genes (DEGs) which were identified by three different methods. **(C)** The volcano map of microarray dataset GSE138206. **(D)** The volcano map of high-throughput sequencing dataset GSE140707. The DEGs are marked in light blue; the 127 common DEGs are labeled with black, and red labels are DEGs in each dataset.

This study was performed according to the workflow (**Figure 1A**). A total of 280 DEGs in microarray dataset GSE138206 were identified by the limma package with 162 upregulated genes and 118 downregulated genes (**Figure 1C**, **Table S1**). Analysis of the high-throughput sequencing datasets GSE140707 by Deseq2 packages obtained 1,699 DEGs of OSCC, and, of these, 652 genes and 1,047 genes were upregulated and downregulated, respectively (**Figure 1D**, **Table S2**). In addition, 1,215 DEGs were identified by EdgeR with 666 upregulated genes and 549 downregulated genes (**Table S3**). Taking the intersection of three different package analyses of two OSCC datasets and plotting the Venn diagram, 127 common DEGs were obtained (**Figure 1B**, **Table S4**). Additionally, there were five significant DEGs (MMP10, MUCL1, TGM3, WIF1, and TMPRSSS11B) that were identified only in GSE138206 (**Figure 1C**). In addition to the intersection, seven high fold change expression genes (CST1, IGHV1-3, IGHV1-18, MAGEA6, HMGCS2, KRT84, and KRTAP13-2) were identified in GSE140707 (**Figure 1D**).

### Dysregulation of Genes Related to Extracellular Matrixes and Cytokines in OSCC

**Figure 2:**
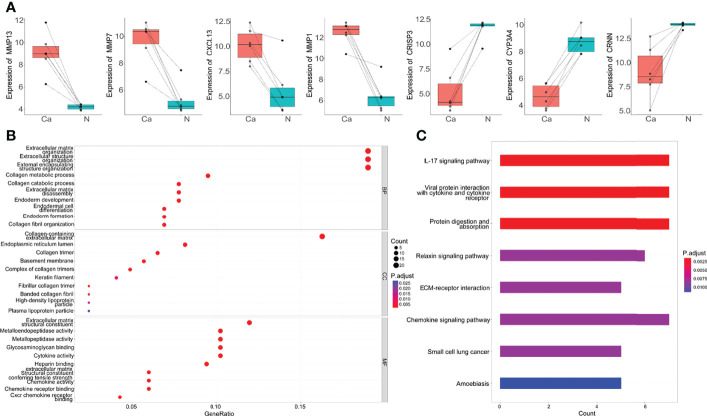
The top DEGs and common DEGs enrichment analysis results. **(A)** The expression of seven DEGs in microarray dataset GSE138206. Ca, cancer; N, Normal. **(B)** The enrichment analysis results of GO database with BP, CC and MF. **(C)** The enrichment analysis results of KEGG Pathway. Adjusted P-value < 0.05 was considered significant.

Seven genes belonged to the intersection of DEGs identified by three different methods were dysregulated genes in OSCC. Among them, MMP13, MMP7, MMP1, and CXCL13 genes were upregulated in OSCC tissues, and CRISP3, CYP3A4, and CRNN genes were downregulated (**Figure 2A**). To gain insight into the pathways and function of common DEGs of OSCC, the GO enrichment analysis and KEGG enrichment analysis were performed. It was observed that DEGs were enriched in categories associated with extracellular matrix (ECM) organization, collagen metabolic process, metallopeptidase activity, glycosaminoglycan binding, and cytokine activity (**Figure 2B**). KEGG analysis showed that the 127 DEGs were significantly enriched in 10 pathways, such as cytokine-cytokine receptor interaction, IL-17 signaling pathway, viral protein interaction with cytokine and cytokine receptor, and protein digestion and absorption. Notably, COL4A1, COL4A2, COL4A6, FN1, and LAMC2 genes were also enriched in the ECM receptor interaction pathway (**Figure 2C**).

### Evolutionary Relationship Between the Dysregulated Genes and Oral Microbiota

**Figure 3:**
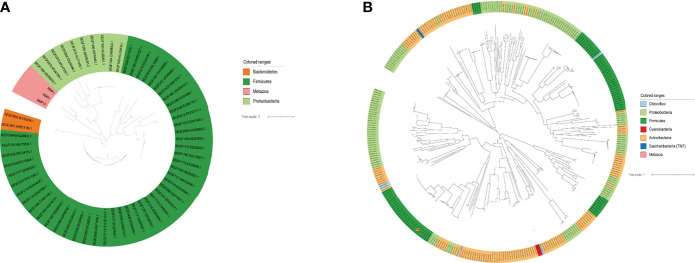
The evolutionary relation analysis of Top DEGs. **(A)** The evolutionary relation between MMP13, MMP7, and MMP1 with the microbiome. **(B)** The evolutionary relation between CYP3A4 with the microbiome.

To explore the evolutionary relationships of the seven DEGs and oral microbiota. We used the BLAST method to align 1,903 microbiome genomes (total 4,665,857 proteins) in human microbiome which retrieved from the eHOMD database. We set the BLAST cutoff with e-value < 10^−3^ against the microbiome genome search for the homologous proteins (**Table 1**). We searched 350 homologous proteins within 45 species of microorganisms with the CYP3A4 gene by BLAST software. Among these microorganisms, *Comamonas testosteroni KF-1*, *Comamonas testosteroni CNB-2*, *Comamonas testosteroni S44*, and *Arachnia rubra DSMZ* 10012 inhabit in the oral cavity (**Table S5**). Oral colonizers with an evolutionary relationship to MMP1 include *Streptococcus infantis ATCC* 700779, *Streptococcus infantis SPAR10*, and *Tannerella* sp.*_HMT_286 W11667* (**Table S6**). Interestingly, *Tannerella* sp.*_HMT_286 W11667* and MMP5 as well as MM13 also have a coevolutionary relationship (**Tables S7**, **S8**). In addition, MMP13 also has a coevolutionary relationship with *Lacticaseibacillus paracasei*, *Lacticaseibacillus rhamnosus*, and *Streptococcus salivarius*, which live in the oral cavity (**Table S8**). **Figure 3A** shows the evolutionary relations among the MMP13, MMP7, and MMP1 genes of oral genomes. As can be seen from the results of the analysis of the evolutionary relationship, three upregulated genes have homologs with the phyla of *Bacteroidetes*, *Firmicutes*, and *Proteobacteria*. The most significant aspect of this relationship is Proteobacteria, which contains 8 oral species and 10 proteins. The evolutionary relations of CYP3A4 genes are shown in **Figure 3B**.

**Table 1:** The homologous proteins searched of seven DEGs.

Gene name	Gene annotation	Species	Strain	Oral	Uassigned	Protein	Species name
CRISP3	cysteine-rich secretory protein (CRISP) family	1	1	0	1	1	NA
CYP3A4	cytochrome P450 superfamily of enzymes	45	119	2	43	350	*Comamonas testosteroni, rubra*
MMP1	peptidase M10 family of matrix metalloproteinases	7	39	4	3	40	*Lacticaseibacillus rhamnosus, Lacticaseibacillus paracasei*, *Streptococcus salivarius, Tannerella* sp.*_HMT_286*
MMP7	peptidase M10 family of matrix metalloproteinases	9	16	1	8	16	*Tannerella* sp.*_HMT_286*
MMP13	peptidase M10 family of matrix metalloproteinases	5	11	2	3	13	*Streptococcus infantis*, *Tannerella* sp.*_HMT_286*
CXCL13	C-X-C Motif Chemokine Ligand 13	0	0	0	0	0	NA
CRNN	The "fused gene" family of proteins	0	0	0	0	0	eNA

## Discussion

OSCC is the most common malignancy, accounting for 80%–90% of oral malignancies. Oral microbiota is a major risk factor for OSCC. Associated interactions between oral microorganisms and host can promote the progression of OSCC (Bai et al., 2022). In this study, we identified 652 upregulated and 1,047 downregulated genes in the OSCC tissues based on the GSE140707 dataset, as well as 162 upregulated and 118 downregulated genes in the GSE138216 dataset. The unique significance of 127 DEGs by three methods was based on the analyzed metadata. DEGs were identified in both datasets associated with the IL-17 signaling pathway, viral protein interaction with cytokines and cytokine receptors, and protein digestion and absorption. Seven significant dysregulated genes in OSCC tissues were further identified, including four upregulated genes MMP13, MMP7, MMP1, and CXCL13 and three downregulated genes CRSP3, CYP3A4, and CRNN. In the present study, we established a predictive pipeline for exploring the evolutionary relationship between the oral microbiota in the eHOMD database and the dysregulated genes in OSCC based on the principle of coevolution.

Matrix metalloproteinases (MMPs) are involved in normal physiological processes of decomposing ECM, such as tissue remodeling, embryonic development, and reproduction, as well as in disease processes of arthritis and metastasis (Snoek-van Beurden and Von den Hoff, 2005). The MMP13, MMP7, and MMP1 genes encode members of the peptidase M10 family of MMPs. Consistent with our findings, previous studies have reported the dysregulated expression of them in OSCC progression. For instance, overexpression of both transcriptional and translational levels of MMP13 was found in OSCC tissues (Johansson et al., 1997; Culhaci et al., 2004; Luukkaa et al., 2006). Highly expressed MMP13 protein also showed a significant correlation with tumor staging and lymph node metastasis (Vincent-Chong et al., 2014). The expression of MMP7 and MMP1 was also upregulated in OSCC tissues. The expression of MMP7 and MMP1 were also upregulated in OSCC tissues. The MMP1 gene was activated in aggressive OSCC (Impola et al., 2004; Jordan et al., 2004; Chuang et al., 2008; Makinen et al., 2014).

Moreover, the MMP1 gene might be used as a potential target to improve diagnosis and as an oral cancer marker for OSCC (Hashimoto et al., 2004; Yen et al., 2009; Yang et al., 2020). Functional enrichment analysis revealed that MMP13, MMP7, and MMP1 were related to ECM organization, ECM disassembly, extracellular structure organization, and endoderm development. Tumors can utilize ECM remodeling to create a microenvironment that promotes tumorigenesis and metastasis. Therefore, we speculate that the overexpression of MMP13, MMP7, and MMP1 might involve invasiveness and metastasis of OSCC by modulating ECM remodeling.

The homology search method identified an evolutionary relationship with the oral microbiota for the MMP13, MMP7, and MMP1 genes. We chose MMP family genes to explore their evolutionary relationship with microbial species because MMP family differential expression was significant when differentially expressed genes were analyzed. The *Lacticaseibacillus paracasei*, *Lactobacillus rhamnosus*, *Streptococcus salivarius*, *Tannerella* sp. *HMT 286*, and *Streptococcus infantis five* species were hit homologous. In the previous study, Pushalkar et al. assessed the microbial diversity in OSCC tissues and non-tumor tissues. The results showed that the microbial load of *Lacticaseibacillus* paracasei has a significant variation (Pushalkar et al., 2012). It has been reported that the administration of *Lactobacillus rhamnosus* was able to increase the effect of anticancer molecules tested on human OSCC (Cheng et al., 2017). These results demonstrated the potential of *Lactobacillus rhamnosus* as a beneficial effect adjuvant treatment for OSCC. In OSCC patients undergoing tumor resection, the percentage of saliva-reactive cytotoxic T cells was positively correlated with recurrence-free survival (Wang et al., 2018). Another study (Pavlova et al., 2013) showed that *Streptococcus salivarius* is involved in alcohol metabolism to acetaldehyde, which has a carcinogenic potential (Vogelmann and Amieva, 2007; Marttila et al., 2013). Consistent with our findings, evidence has also shown a significant difference of *Streptococcus infantis* between OSCC patients and healthy individuals (Hsiao et al., 2018). These all support the reliability of our predictive pipeline for exploring the interaction between dysregulated genes in OSCC and oral microbiota.

The CYP3A4 gene encodes a member of the cytochrome P450 superfamily of enzymes and is involved in the metabolism of sterols, steroid hormones, retinoids, and fatty acids (Chen et al., 2000; Marill et al., 2000; Badawi et al., 2001). Cytoscape software by the Matthews correlation coefficient (MCC) algorithm was used to predict CYP3A4 at the core position in the network and highlight the first 10 types of OSCC DEGs (Li et al., 2022). It is worth noting that there found 350 homologous proteins including 45 microbes were found by evolutionary analysis in this study. Among them, *Comamonas testosteroni* and *Arachnia rubra* can inhabit in the oral cavity. Up to 348 microorganisms were unassigned information on their location in the human body. Extracts of *Arachnia rubra* are associated with human OSCC production and modulation of tumor-specificity values (Suzuki et al., 2014). However, we noticed that the two previously reported bacteria associated with OSCC, *Porphyromonas gingivalis* and *Clostridium perfringens*, were not found when using this method to explore the relationship between dysregulated genes and oral microbiota. We speculate that the possible reason is that, although these bacteria play an important role in OSCC, there is no homologous evolutionary relationship with the genes that we screened. CXCL13, CRISPO3, and CRNN have newly identified dysregulated genes by this study, and their effects on the OSCC need to be further investigated for experimental validation.

Microorganisms, including bacteria, viruses, and archaea, inhabit a wide range of hosts in different ecological niches and ecosystems (Braga et al., 2016). Deciphering microbial–host interactions can provide new therapeutic strategies for maintaining health or improving disease states. However, determining microbial–host interactions between species experimentally is a challenging task due to many other limitations related to the size, scope, feasibility of studies, and sample availability of microbial populations (Fritz et al., 2013). Computational approaches can overcome some of these limitations and thus enhance our understanding of microbial–host interactions (Dix et al., 2016). Molecular ecological networks are used to study the interactions between molecules (from different species or even kingdoms) in a larger ecosystem (Yang et al., 2017; Meyer et al., 2020). From a mechanistic perspective, the most widely studied types of interactions among species interactions include microbial networks, PPIs, and RNA-mediated interactions. Therefore, many computational methods developed to study microbe–host interactions have focused on the three types of interactions mentioned above. However, all of these inference methods have the feature of studying microbe–host interactions based on the characteristics of the sequence structure. In this study, we propose the use of coevolutionary principles to infer microbial–host interactions based on sequence structure. The use of the coevolutionary principle better reflects the conserved protein structure of the species than the direct use of sequence structure. Therefore, we established a predictive pipeline to study the interaction between DEGs and oral microbiota in OSCC based on sequence-structure conservativeness and coevolutionary principles. Of course, the method has its shortcomings, that is, the method is based on the inference that species have the same protein conserved modules during the evolutionary process, and if the studied DEGs and microbial proteins do not have the same evolutionary conserved modules, they cannot be studied by this method. However, this method provides a novelty way and new ideas for exploring the relationship between host genes and host symbiotic microorganism.

## Data Availability Statement

The original contributions presented in the study are included in the article/**Supplementary Material**. Further inquiries can be directed to the corresponding author.

## Author Contributions

YF contributed to the study conception and design, data acquisition, analysis, and interpretation, and in the drafting the of the manuscript. YF and YY contributed to data acquisition, analysis, and interpretation. CL contributed to the study conception and design and the drafting and critical revision of the manuscript. All the authors approved the final version of the manuscript and agreed to be accountable for all aspects of the work.

## Funding

This work is supported by PhD research startup foundation of the Third Affiliated Hospital of Zhengzhou University (2021080).

## Conflict of Interest

The authors declare that the research was conducted in the absence of any commercial or financial relationships that could be construed as a potential conflict of interest.

## Publisher’s Note

All claims expressed in this article are solely those of the authors and do not necessarily represent those of their affiliated organizations, or those of the publisher, the editors and the reviewers. Any product that may be evaluated in this article, or claim that may be made by its manufacturer, is not guaranteed or endorsed by the publisher.

## Supplementary Material

Supplementary Table 1 The DEGs in GSE138206 dataset were identified by limma packages.

Supplementary Table 2 The DEGs in GSE140707 dataset were identified by the Deseq2 package.

Supplementary Table 3 The DEGs in GSE140707 dataset were identified by the EdgeR package.

Supplementary Table 4 The 127 common DEGs.

Supplementary Table 5 The homolog searched results of CYP3A4 genes.

Supplementary Table 6 The homolog searched results of MMP13 genes.

Supplementary Table 7 The homolog searched results of MMP7 genes.

Supplementary Table 8 The homolog searched results of MMP1 genes.

## Abbreviations

DEGs, differentially expressed genes; MMPs, peptidase M10 family of matrix metalloproteinases; OSCC, oral squamous cell carcinoma; GEO, Gene Expression Omnibus; GO, The Gene Ontology; MF, Molecular Function; CC, Cellular Component; BP, Biological Process; FDR, adjusted P-value; FC: fold change; KEGG, Kyoto Encyclopedia of Genes and Genomes.
